# The Role of Semaphorins and Their Receptors in Innate Immune Responses and Clinical Diseases of Acute Inflammation

**DOI:** 10.3389/fimmu.2021.672441

**Published:** 2021-05-03

**Authors:** Shreya M. Kanth, Salina Gairhe, Parizad Torabi-Parizi

**Affiliations:** Critical Care Medicine Department, Clinical Center, National Institutes of Health, Bethesda, MD, United States

**Keywords:** semaphorin, plexin, neuropilin, innate immunity, sepsis, acute inflammation

## Abstract

Semaphorins are a group of proteins that have been studied extensively for their critical function in neuronal development. They have been shown to regulate airway development, tumorigenesis, autoimmune diseases, and the adaptive immune response. Notably, emerging literature describes the role of immunoregulatory semaphorins and their receptors, plexins and neuropilins, as modulators of innate immunity and diseases defined by acute injury to the kidneys, abdomen, heart and lungs. In this review we discuss the pathogenic functions of semaphorins in clinical conditions of acute inflammation, including sepsis and acute lung injury, with a focus on regulation of the innate immune response as well as potential future therapeutic targeting.

## Introduction

The semaphorins (SEMA) are a large family of both membrane-bound and secreted proteins defined by the presence of an approximately 500-amino acid extracellular Sema domain located at the N- terminal region (1). Although semaphorins contain the Sema domain, other human proteins also can contain this domain (2). Members of the semaphorin family are subdivided into eight classes, classes 1-7 and V, based on specific domains in their carboxy-terminal regions and phylogenetic tree analysis. Classes 1 and 2 are only found in invertebrate species, classes 3-7 in vertebrates, and class V members are found in DNA viruses, including the viral semaphorin A39R (3) (**Supplementary Table 1**). Differing semaphorin classes vary in their membrane anchorage, including secreted and membrane-associated forms. Membrane-associated semaphorins exist as transmembrane semaphorins or linked to glycosylphosphatidylinositol (GPI). Class 2, 3, and V semaphorin members are all secreted, whereas classes 4–6 are all transmembrane proteins, and SEMA7A is linked to the plasma membrane *via* a GPI anchor (4–7). Notably, some of the primarily membrane-associated semaphorins may be cleaved to produce soluble proteins. Proteolytic cleavage of semaphorins to a soluble form allows for diffusion locally and systemically to various effector cells, including immune cells. More recently, a soluble form of semaphorin SEMA5A was described in the context of both pancreatic cancer and rheumatoid arthritis (8, 9). Additionally, SEMA4A and SEMA4D have been found to exist as soluble proteins after proteolytic cleavage mediated by matrix metalloproteinases (10–14).

Activation of downstream signaling pathways involving semaphorins is dependent on the binding of semaphorins to their receptors, resulting in “forward signaling”. Plexins (PLXN) and neuropilins (NRP) are the predominant receptors for semaphorins (15–17), and nine plexins (PLXNA1–PLXNA4, PLXNB1–PLXNB3, PLXNC1 and PLXND1) and two neuropilins (NRP1, NRP2) have been identified in vertebrates. Several other proteins have also been identified as participating in semaphorin signaling, including integrins, T cell immunoglobulin and mucin domain-containing protein 2 (TIM2), and CD72. Furthermore, additional co-receptors have been found to be in close association with plexins and neuropilins on the cell surface. For example, association of adhesion molecule L1-CAM with NRP1 functions as a receptor for SEMA3A and semaphorins can transactivate plexin-associated receptor tyrosine kinases OTK/PTK7, MET, RON, VEGFR2, and ERBB2 (18–23). Although the molecular mechanisms that are responsible for the varying effects of the semaphorin-plexin complex are not fully understood, early studies suggest that activation of Ras and Rho GTPases by plexin GAP has been shown to play a crucial role in cellular proliferation, adhesion and migration (4). Interestingly, transmembrane semaphorins can also function as receptors on cells expressing them to initiate “reverse signaling”. SEMA6D can act as a receptor and upon interaction with a tyrosine kinase, c-Alb, it triggers anti-inflammatory macrophage polarization by controlling fatty-acid metabolism (24). Similarly, binding of PLXNB1 to SEMA4A, which is present as a receptor on dendritic cells, has been shown to regulate migration (25). Beside this, recent studies suggest that semaphorins can act as a co-stimulatory molecule and aid in immune cell activation and regulation of the immune response. For example, dendritic cells (DC) express SEMA3A and inhibit T cell activation whereas SEMA4A binds to Tim-2 on T cells resulting in activation and proliferation. SEMA4D additionally indirectly co-stimulates T cell activity (26). Human monocyte-derived DCs express high levels of SEMA4A which through its newly identified receptor immunoglobulin-like transcript 4 (ILT-4) on CD4+ T cell was shown to stimulate T cell differentiation (10). Additionally, the high evolutionary conservation of semaphorins and plexins among different species suggests that they have fundamental roles in the regulation of multiple cellular functions (27).Taken together, these data suggest that semaphorins can trigger multiple signaling pathways in a wide range of cellular contexts to carry out distinct biological activities, including activation and regulation of immune responses.

Although semaphorins were originally identified for their involvement in neural guidance and development, they have since been found to have important roles in many pathophysiological processes. Work over the past 20 years has demonstrated the function of semaphorins and their receptors in numerous diseases including cancer (28–31), disorders of bone turnover (32–37), angiogenesis (38–42), autoimmune and rheumatologic diseases (43–50), and inflammatory conditions. In this Review, we summarize the rapidly evolving literature on the functional roles of immune semaphorins at a cellular and intercellular level, and discuss their pathogenic roles and therapeutic implications in acute inflammatory diseases and in the innate immune response.

## Cellular Function of Semaphorins and Their Receptors in Acute Inflammation

**Figure 1:**
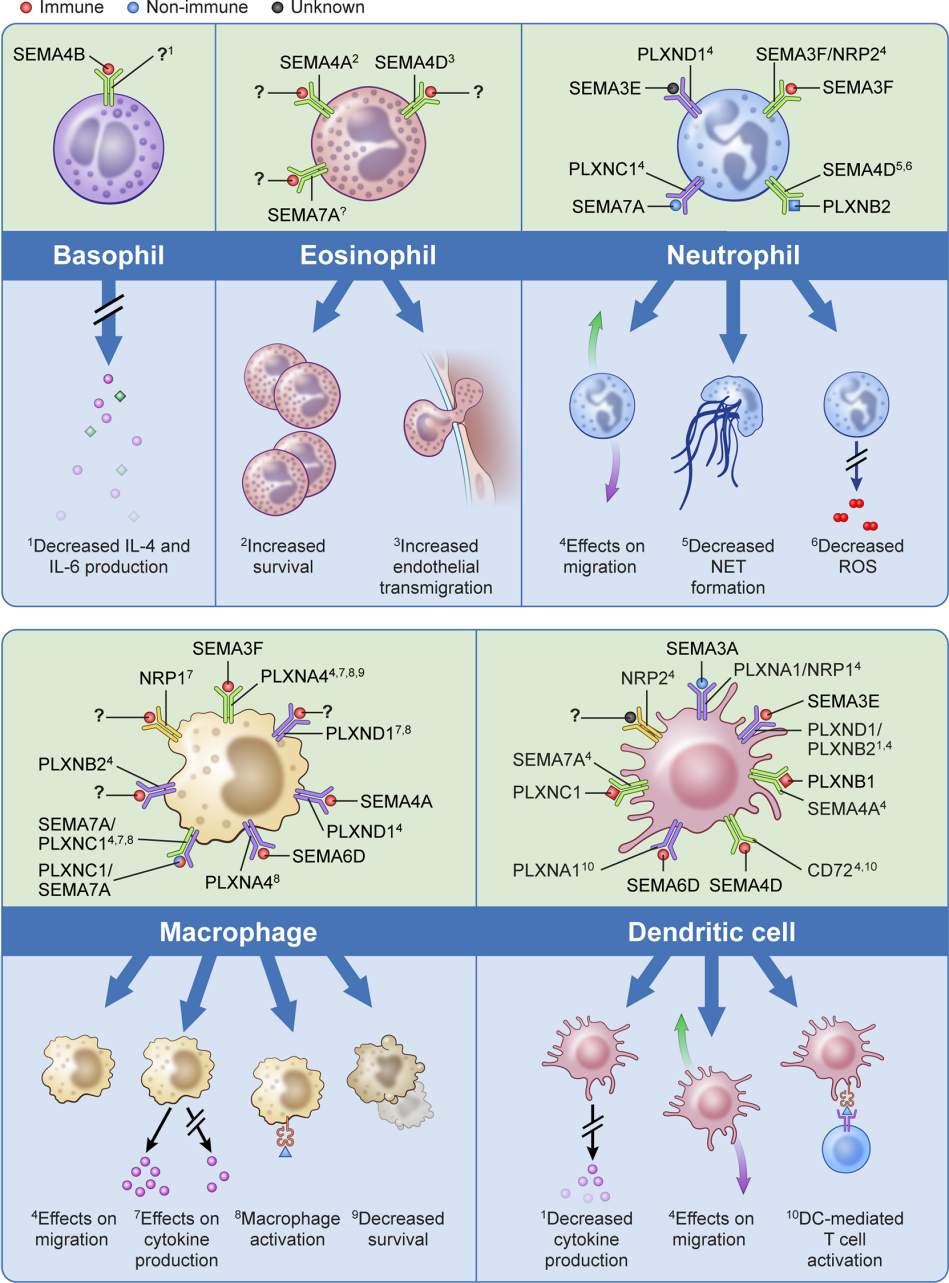
The effects of semaphorins and their receptors on cells of the innate immune system. Semaphorins and their binding partners in relation to innate immune cells are shown here. Ligands depicted in red have been shown to originate from immune cells, those in blue from non-immune cells, and for those depicted in black the cell of origin is unknown.? indicates unknown ligand for the depicted receptor. Superscripts are related to the processes described in the lower part of the panels for each cell type;^?^ indicates that a specific function has not been associated with the interaction shown.

Over the past decade there has been significant evolution in our understanding of immune semaphorins, with a focus on their involvement in the adaptive immune response (51). However, more recent studies have investigated their role in innate immunity including the recruitment and migration of immune cells, modulation of the function and phenotype of myeloid cells, regulation of proinflammatory cytokines, and immune cell survival (**Figure 1**).

Cell migration is a fundamental process in the immune response, and extracellular guidance cues play a key role in directing this movement in an orchestrated fashion. Semaphorins have been shown to act either as attractive or repulsive cues for migration of neutrophils, macrophages, dendritic cells and eosinophils (13, 39, 44, 52–66). Structurally, both semaphorins and plexins are characterized by the presence of plexin-semaphorin-integrin (PSI) domains adjacent to the N-terminus Sema domain, and plexins also contain a GTPase-activating protein (GAP) domain and a Rho GTPase-binding domain. As such, it is thought that the binding of semaphorins to plexins activates GTPases which modulates integrin-mediated cell attachment, actomyosin contraction, and microtubule destabilization which all may influence cellular migratory abilities (22, 67, 68). For example, treatment of human peripheral blood neutrophils with exogenous SEMA3F has been shown to inhibit neutrophils from migrating towards a chemoattractant in Transwell assays. This slowing of neutrophil migration as well as increased neutrophil rounding is mediated by F-actin reorganization in a zebrafish model of inflammation (57). Conversely, exogenous SEMA3A markedly reduced neutrophil recruitment into the lungs in mouse models of airway allergen exposure (55). This highlights class and subclass differences in semaphorins may have varying and opposing effects on immune cell migration.

Macrophages are key immune effector cells in the innate immune response. They have a remarkable ability to change their phenotype following exposure to pro- or anti-inflammatory stimuli. The semaphorin-plexin axes in the presence or absence of these stimuli have shown to be potent monocyte and macrophage activators with different semaphorin classes giving rise to specific macrophage populations (13, 24, 39, 69–72). For example, while SEMA3A promotes the transition of classically activated (M1) macrophages towards a resolution phenotype, SEMA3E positively regulates macrophage activation towards M1 phenotype (71, 72).

In addition to their aforementioned functions, the expression of semaphorins and their receptors on various innate immune cells plays a role in the release of pro-inflammatory cytokines as well as in innate immune cell survival (44, 50, 73–80). This suggests potential targeted therapies involving semaphorins, plexins, and neuropilins may promote the resolution phase of acute inflammation and mitigate tissue and end-organ damage from inflammatory conditions, such as sepsis and ARDS.

## Role of Semaphorins and Their Receptors in Clinical Syndromes

**Figure 2:**
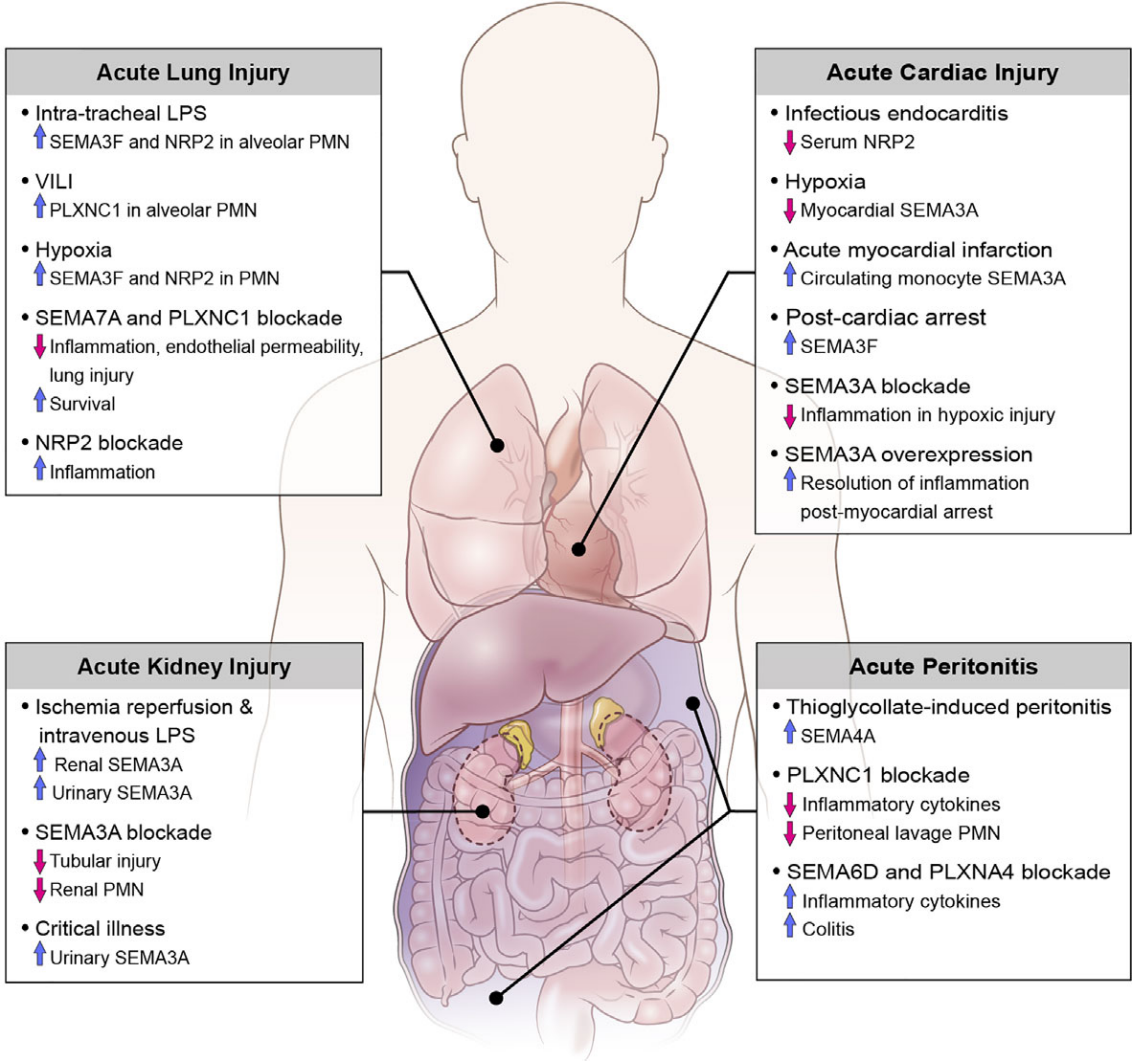
Role of semaphorins and their receptors in clinical conditions of acute inflammation.

Translational studies and investigations using animal disease models have demonstrated the involvement of various classes of semaphorins, plexins, and neuropilins as both pro- and anti-inflammatory mediators in the pathogenesis of acute inflammation. Here we discuss the role of semaphorins in acute inflammatory clinical syndromes including sepsis, as well as diseases defined by injury to the kidneys, abdomen, heart and lungs (**Figure 2**).

## Sepsis and Endotoxin-Induced Inflammation

Sepsis is a complex inflammatory syndrome characterized by a dysregulated host response to infection resulting in life-threatening organ dysfunction (81). The initial activation of the innate immune response occurs *via* binding of pathogen- associated molecular patterns (PAMPs), such as lipopolysaccharide (LPS) or bacterial endotoxin, to specific receptors resulting in activation of downstream signal transduction pathways, and ultimately to both pathogen and host cell and tissue damage (82).

Mouse models of sepsis by means of intraperitoneal injection of LPS reveal the role of SEMA3A, SEMA3E and their receptors PLXNA4 and PLXND1, respectively, in propagating the inflammatory cascade. Congruously, inhibition of these semaphorins and their receptors is associated with an attenuated septic response including reduced levels of proinflammatory cytokines, lower clinical scores of sepsis, and improved survival rates (71, 79, 83). For example, Wen et al. (79) demonstrate a critical role for SEMA3A and its ligand PLXNA4 in the activation and downstream signaling of the Toll-like receptor (TLR) pathways in clinical sepsis. By studying PLXNA4- deficient mice, they show that TLR4 signaling is attenuated by means of decreased inflammatory cytokine production, including TNF-α and IL-6, through intersection of PLXNA4 and TLR4 with the activation of Rac1 and JUN N-terminal kinase (JNK)-mediated nuclear factor- κB (NF-κB) activation. Furthermore, Yamashita et al. (83) explore the application of an anti-SEMA3A neutralizing antibody in mouse models of sepsis which results in increased survival after four days. Although there are no translational studies investigating the use of semaphorins to treat sepsis in humans, these studies suggest an avenue for further research and ultimately a clinical application.

On the contrary, NRP1, which has been identified as a co-receptor for SEMA3A, is shown to down-regulate the systemic inflammatory response of sepsis. In fact, in cecal ligation and puncture as well as intraperitoneal LPS models of sepsis using myeloid cell-specific NRP1-knockout mice there is increased production of pro-inflammatory cytokines (iNOS, TNF-α, IL-6) in peritoneal lavage and serum and higher mortality compared with wild-type mice (73). This suggests that there are differing roles of semaphorins and their receptors within the same class in the regulation of septic inflammation.

## Acute Kidney Injury

Previous investigations have demonstrated that SEMA3A is expressed within the developing and mature kidney, specifically in the distal tubules and collecting tubules (84). More recently, reports of acute kidney injury (AKI) in mouse models of sepsis and ischemia-reperfusion describe the pathogenic role of semaphorins in the promotion of tubular epithelial cell injury and restoration of renal function. In mice treated with intra-peritoneal LPS for up to 24 hours, renal histopathology shows increased neutrophil and macrophage infiltration with apoptosis of tubular epithelial cells (TECs) and increased SEMA3A expression in proximal TECs; this injury is attenuated when SEMA3A is inhibited by (−)-epigallocatechin-3-gallate (EGCG) (85). After ischemia-reperfusion injury of the kidneys, a large increase in urinary and renal tissue SEMA3A is detected. Notably, genetic inactivation or functional inhibition of SEMA3A is renoprotective against ischemia-reperfusion renal injury in animal models revealing blunted expression of inflammatory cytokines, reduced renal neutrophil infiltration, and decreased tubular cell apoptosis (86). In a translational single-center prospective cohort study of critically ill patients admitted to an intensive care unit, levels of urinary SEMA3A on admission were significantly higher in patients with AKI compared to non-AKI patients. Additionally, urinary SEMA3A levels were significantly higher in patients with late-onset AKI and AKI progression as compared to established AKI groups and non-AKI progressors. However, urinary SEMA3A was not reflective of AKI severity and there was no difference in levels of SEMA3A in septic versus non-septic patients (87). Although there are many limitations to this study, it is important to recognize the potential use of semaphorins as biomarkers for various disease processes associated with critically ill patients, and further studies to investigate their role in septic shock-related end organ dysfunction are warranted.

## Acute Peritonitis

In investigations of animal models with localized acute inflammation, as in with acute peritonitis, SEMA7A and its receptor PLXNC1, as well as SEMA4A are shown to enhance the inflammatory response. Murine models of peritonitis induced by thioglycolate demonstrate the upregulation of SEMA4A on peritoneal myeloid precursors (CD11b+ and CD68+ cells), and SEMA4A is significantly upregulated specifically in inflammatory subsets of circulating monocytes (Ly6C^high^) whose function is to selectively traffic to sites of tissue damage (39). Similarly, in zymosan A (ZyA)- induced peritonitis in mice, genetic inactivation and functional inhibition of PLXNC1 results in reduced peritoneal lavage granulocyte counts, protein content, MPO activity, and cytokine levels (TNF-a, IL-6, MIP-1-a, MIP-2). Furthermore, inactivation of PLXNC1 demonstrates increased leukocyte rolling, reduced adhesion and reduced infiltration into the parietal peritoneum perivascular tissue compared to wild-type mice, resulting in the dampened acute inflammatory response (88). Conversely, SEMA6D and its receptor PLXNA4 have been shown to play a critical role in the polarization of macrophages into anti-inflammatory states, and deficiency of SEMA6D in mice results in increased inflammatory cytokine production by peritoneal macrophages as well as predisposition to colitis after intraperitoneal administration of LPS and dextran sodium sulfate (DSS) (24). Overall, these findings demonstrate class specific responses of various semaphorins in the pathogenesis of acute peritonitis and may reveal opportunities for novel therapeutics.

## Acute Cardiac Dysfunction

Semaphorins have previously been shown to play critical functions in cardiogenesis and blood vessel development (89), however more recently they have been explored as biomarkers for various inflammatory cardiovascular conditions in critically ill patients. In a study of patients with clinical suspicion of infectious endocarditis (IE) the investigators performed proteomic analysis of serum to identify putative biomarkers of IE by means of gel electrophoresis and mass spectrometry. Overall, 7 proteins present in at least 70% of all samples were differentially regulated in those with confirmed IE, with neuropilin 2 (NRP) significantly downregulated in IE patients. In models of hypoxia-induced myocardial inflammation SEMA3A levels are decreased in myocardiocytes, and further silencing of the *SEMA3A* gene is associated with reduced myocardial cell injury after hypoxia and increased resolution of inflammation by means of decreased inflammatory cytokines, decreased cardiomyocyte apoptosis and increased cell viability (90). On the contrary, after myocardial infarction there is increased expression of SEMA3A on circulating monocytes, and stimulation of classically activated monocytes with recombinant SEMA3A promotes their transition towards a resolution phenotype. Additionally, SEMA3A heterozygous mice showed worse cardiac function with decreased fractional shortening of the heart compared to wild type littermates (72). A study of patients who survived out-of-hospital cardiac arrest revealed higher serum SEMA3F levels compared to stable patients with coronary artery disease and healthy volunteers, as well as significantly elevated SEMA3F levels in patients with severe myocardial dysfunction (left ventricular ejection fraction less than 40%) compared to those with normal or mildly reduced ejection fraction. Subgroup analysis noted that elevated SEMA3F levels after return of spontaneous circulation is associated with decreased survival, myocardial dysfunction, as well as prolonged vasopressor therapy (91). More recently, Köhler et al. (92) explored the novel expression and function of SEMA7A in myocardial ischemia-reperfusion injury (MIRI). They demonstrate the increased expression of soluble SEMA7A in patients with acute myocardial infarction associated with a rise in platelet-neutrophil complexes (PNC). Given the hypothesis that SEMA7A plays a pivotal role in the progression of MIRI, the investigators demonstrated that administration of SEMA7A results in increased MIRI and PNC formation and this pathology is due to an interaction of SEMA7A with platelet glycoprotein Ib in animal models. Importantly, they also show that administration of a function-blocking anti-SEMA7A antibody before the start of reperfusion resulted in decreased infarct size and reduced troponin level. These findings have important implications towards the development of treatments that target thrombo-inflammatory MIRI, and further studies to validate these findings are clinically valuable.

## Acute Lung Injury

Acute lung injury (ALI) and acute respiratory distress syndrome (ARDS) describe syndromes of high morbidity and mortality in critically ill patients. The clinical hallmarks include acute onset of respiratory failure with varying degrees of hypoxemia, bilateral infiltrates as seen on chest radiograph, and the absence of cardiogenic pulmonary edema. From a more conceptual standpoint, a plethora of predisposing insults, such as sepsis, trauma, and inhalation of noxious stimuli, leads to inflammation in the lungs with resulting increased pulmonary vascular permeability and alveolar collapse. Pathological hallmarks of ALI are characterized by injury to the alveolar epithelium and endothelium, coagulopathy and activation of the innate immune response, which further amplifies lung damage (93–96). Semaphorins have emerged as key mediators of multiple mechanisms contributing to ALI including neutrophil migration, vascular permeability, cytoskeletal remodeling and the initiation of inflammatory signaling cascades, which we review here.

Neutrophils play a major role in ALI progression in both human and animal studies, and activation and transmigration of neutrophils can lead to tissue damage by release of cytotoxic and immune-cell activating mediators (97–100). SEMA3F and SEMA7A have emerged as regulators of neutrophil migration in models of acute lung injury. In response to intratracheal LPS challenge in a murine model, SEMA3F is released into the airways, with neutrophils from bronchoalveolar lavage expressing SEMA3F and its receptor NRP2 at both the mRNA and protein levels. Neutrophil-specific loss of SEMA3F results in more rapid neutrophil recruitment to and clearance from the lungs in this model, without evidence of increased neutrophil apoptosis. Concurrently, exogenous intratracheal administration of SEMA3F results in increased recovery of neutrophils in BAL and alveolar space. Evaluation of live lung slices from mice stimulated with SEMA3F demonstrates reduced neutrophil speed, lower F-actin content, and increased rounding of neutrophils, all of which likely contribute to retention of neutrophils at sites of inflammation (57). Additionally, SEMA7A induces transendothelial migration of neutrophils into lung tissue in conditions of hypoxia and after LPS inhalation (54, 60), and blockade of SEMA7A in both *in vivo* and *in vitro* models reveals attenuated injury-induced influx of neutrophils correlating with dampened lung injury (54, 60, 101). This action is dependent on binding of SEMA7A to the PLXNC1 receptor (53, 54), and animal models of ventilator-induced lung injury (VILI) demonstrate robust induction of PLXNC1 on neutrophils. Furthermore, *in vivo* inhibition of SEMA7A and PLXNC1 by use of neutralizing antibodies in models of VILI and inhaled LPS models results in improved survival, reduced neutrophil migration and overall reduced cytokine response and lung injury (53, 101), suggesting their use a novel therapeutic agent for the management of ALI.

The pulmonary endothelium serves as a semipermeable barrier between the pulmonary circulation and the interstitium, and disruption of the integrity of this barrier by means of cytoskeletal remodeling leads to worsening pulmonary edema, impaired gas exchange, and release of pro-inflammatory markers and coagulation factors (102). In models of ALI from seawater aspiration, inhibition of SEMA7A and its receptor PLXNC1 results in a significant reduction in actin-dependent cytoskeletal remodeling and decreased monolayer permeability in rat pulmonary microvascular endothelial cells (80, 103).

Lastly, semaphorins and their receptors have been implicated in the regulation of inflammatory signaling cascades and the release of immune cytokines and chemokines in settings of ALI. Cell surface and soluble NRP2 expression in murine alveolar macrophages is dramatically increased following LPS inhalation, and myeloid-specific ablation of NRP2 results in prolonged accumulation of airway neutrophils and macrophages, as well as increased expression of chemokine (C-C motif) ligand 2 (Ccl2) expression in the lungs with ALI, suggesting NRP2 expression suppressed inflammatory responses to inhaled LPS (104). On the contrary, SEMA7A and its receptor PLXNC1 both induce the release of inflammatory cytokines in various models (53, 60, 80, 103). Taken together, this demonstrates that differing classes of semaphorins and their receptors play roles in both the induction and suppression of the innate immune response and inflammatory cascade in ALI.

## Future Prospects

Semaphorins and their receptors have an important role in the pathophysiology of acute inflammation, and they serve as potential therapeutic targets in several clinical diseases, as summarized above. To date, preclinical animal models have demonstrated the targeting of semaphorins and plexins in cancer, bone diseases, autoimmune diseases, microvascular diseases, allergic airway disease, and in central nervous system regeneration (7). While there are a currently a number of clinical trials underway targeting semaphorins in the context of cancer (clinicaltrials.gov, NCT 03690986, NCT 03373188, NCT 01313065, NCT 03268057), multiple sclerosis (clinicaltrials.gov, NCT 01764737), and Alzheimer’s Disease (clinicaltrials.gov, NCT 04381468), there is only one observational clinical trial investigating peripheral blood semaphorin levels in patients with early septic shock (clinicaltrials.gov, NCT 02692118), and there are no clinical trials investigating targeting semaphorins in conditions of acute inflammation. As highlighted in this review, the most promising areas for the development of therapeutics directed at semaphorins and their receptors in this field of study are sepsis (79), myocardial ischemia (92), and acute lung injury (53–55, 57, 60). Further elucidation of the expression and function of semaphorins and their receptors on innate immune cells will be important in determining additional prospective use as diagnostic or therapeutic targets.

## Author Contributions

SK, SG, and PT-P all contributed to the conception and design of this manuscript. SK contributed to drafting this manuscript. SK, SG, and PT-P all contributed to revisions of this manuscript. All authors contributed to the article and approved the submitted version.

## Funding

Intramural funding from the National Institutes of Health supported this work.

## Conflict of Interest

The authors declare that the research was conducted in the absence of any commercial or financial relationships that could be construed as a potential conflict of interest.

## Abbreviations

SEMA, semaphorin; PLXN, plexin; NRP, neuropilin.
