# Supplementary material for: The Role of Semaphorins and Their Receptors in Innate Immune Responses and Clinical Diseases of Acute Inflammation
Source: Front Immunol. 2021 May 3;12:672441. doi: 10.3389/fimmu.2021.672441 (PMC8126651; doi:10.3389/fimmu.2021.672441)
Supplement: Supplementary file 1 [file Table_1.docx]

| **Class** | **Semaphorins** | **Receptors** |
| --- | --- | --- |
| **III** | SEMA3A | PLXNA1-4, PLXND1, NRP1, L1CAM |
|  | SEMA3B | PLXNA, NRCAM, NRP1, NRP2 |
|  | SEMA3C | PLXNA1-2, PLXND1, NRP1, NRP2 |
|  | SEMA3D | PLXNA1-4, NRP1 |
|  | SEMA3E | PLXNA1-4, PLXNB2, PLXND1, NRP1 |
|  | SEMA3F | PLXNA1-4, NRCAM, NRP1, NRP2 |
|  | SEMA3G | PLXNA1-4, NRP2 |
| **IV** | SEMA4A | PLXNB1-3, PLXND1, TIM2, NRP1, ILT-4 |
|  | SEMA4B | PLXNB1-2, NMDAR |
|  | SEMA4C | PLXNB2, PLXNC1, CD72 |
|  | SEMA4D | PLXNB1-2, PLXND1, CD72 |
|  | SEMA4E | PLXNB1-2 |
|  | SEMA4F | No receptor reported |
|  | SEMA4G | PLXNB2 |
| **V** | SEMA5A | PLXNA1, PLXNA3, PLXNB3, Syn3, MET |
|  | SEMA5B | PLXNA1, PLXNA3, TAG-1 |
| **VI** | SEMA6A | PLXNA2, PLXNA4 |
|  | SEMA6B | PLXNA2, PLXNA4 |
|  | SEMA6C | PLXNA1 |
|  | SEMA6D | PLXNA1, PLXNA4, Trem2, DAP12 |
| **VII** | SEMA7A | PLXNC1, α1β1 Integrin |

DAP12, DNAX-activation protein 12; L1CAM, neural cell adhesion molecule L1; MET, Mesenchymal Epithelial Transition ; NRCAM, neuronal cell adhesion molecule; NMDAR, N-methyl-D-aspartate receptor; NRP, neuropilin; SEMA, semaphorin; Syn3, Synapsin-3; TAG-1, transient axonal glycoprotein 1; TIM2, T‑cell immunoglobulin and mucin domain 2

**Supplementary Table 1: Mammalian adult semaphorins and receptors**
